# Lightweight tea bud detection method based on improved YOLOv5

**DOI:** 10.1038/s41598-024-82529-x

**Published:** 2024-12-28

**Authors:** Kun Zhang, Bohan Yuan, Jingying Cui, Yuyang Liu, Long Zhao, Hua Zhao, Shuangchen Chen

**Affiliations:** 1 College of Physics and Electronic Engineering, Xinyang Normal University, Xinyang, 464000 China; 2 College of Agricultural Equipment Engineering, Henan University of Science and Technology, Luoyang, 47100 China; 3 College of Horticulture and Plant Protection, Henan University of Science and Technology, Luoyang, 47100 China

**Keywords:** Lightweight model, Tea bud detection, YOLOv5, EfficientNetV2, Plant sciences, Engineering

## Abstract

Tea bud detection technology is of great significance in realizing automated and intelligent plucking of tea buds. This study proposes a lightweight tea bud identification model based on modified Yolov5 to increase the picking accuracy and labor efficiency of intelligent tea bud picking while lowering the deployment pressure of mobile terminals. The following methods are used to make improvements: the backbone network CSPDarknet-53 of YOLOv5 is replaced with the EfficientNetV2 feature extraction network to reduce the number of parameters and floating-point operations of the model; the neck network of YOLOv5, the Ghost module is introduced to construct the ghost convolution and C3ghost module to further reduce the number of parameters and floating-point operations of the model; replacing the upsampling module of the neck network with the CARAFE upsampling module can aggregate the contextual tea bud feature information within a larger sensory field and improve the mean average precision of the model in detecting tea buds. The results show that the improved tea bud detection model has a mean average precision of 85.79%, only 4.14 M parameters, and only 5.02G of floating-point operations. The number of parameters and floating-point operations is reduced by 40.94% and 68.15%, respectively, when compared to the original Yolov5 model, but the mean average precision is raised by 1.67% points. The advantages of this paper’s algorithm in tea shot detection can be noticed by comparing it to other YOLO series detection algorithms. The improved YOLOv5 algorithm in this paper can effectively detect tea buds based on lightweight, and provide corresponding theoretical research for intelligent tea-picking robots.

## Introduction

Xinyang is located at the junction of three provinces of Hubei, Henan, and Anhui, in the south of Henan Province, between the northern foot of the Dabie Mountains and the upper reaches of the Huaihe River, with unique natural conditions, and at the same time created the excellent quality of Xinyang Maojian. In recent years, with the booming development of Xinyang Maojian, the lack of labor has gradually become the biggest obstacle to the development of Xinyang Maojian. Xinyang Maojian planting areas are basically in the mountains and hills above, there are currently two ways of mechanical picking and manual picking, while most of the mechanical picking is non-selective picking, followed by the need for manual screening and sorting. Therefore, the development of intelligent tea-picking machinery can effectively alleviate the increasing shortage of tea-picking labor, but also the urgent need for the development of the tea industry^1^.

The prerequisite for intelligent tea picking is the need to accurately identify tea buds in the tea garden. In recent years, many excellent detection algorithms have emerged in the field of tea bud detection, but the current mainstream method is to use deep learning models to detect tea buds^2–5^. Xu et al.^6^ used the Faster R-CNN deep network model for tea bud recognition, showing that the deep learning model can effectively recognize the tea buds. Zhu et al.^7^ further explored the effect of the Faster R-CNN model in detecting tea buds in complex backgrounds and tested the model’s detection accuracy for different types of tea buds, indicating that the deep learning detection algorithm is superior to traditional detection algorithms in terms of detection accuracy and speed in complex backgrounds. Xu et al.^8^ integrated the YOLOv3 detection network with the DenseNet201 model to realize the accurate detection of tea shoots, and compared the effect of shoot detection under two different viewing angles, namely side-view, and top-view, and concluded that the accuracy of this method in detecting tea shoots under the side-view angle was 10.6% higher than that under the top-view angle. Lyu et al.^9^ to solve the problem of the high-brightness image in the process of tea bud detection will lead to more missed detection, proposed a YOLOv5-based tea buds detection model, the realization of the brightness of the image region adaptive correction, and has a strong robustness to the change of light intensity. Wang et al.^10^ improved the detection accuracy of the model for tea buds by improving the spatial pyramid pooling fast in the backbone network of the YOLOv5s model, and at the same time, introduced a bidirectional feature pyramid network and a convolutional block attention module in the neck network to improve the model’s detection accuracy for tea buds, and the experimental results concluded that the improved model outperforms the original model in terms of precision, recall, and mean average precision by 4.4, 0.5, and 4% points, respectively.

Although algorithms based on deep learning have made great strides in the field of tea bud recognition, there are still significant problems in practical scenarios. It is well known that deep learning algorithms require high-performance hardware devices to complete both the model training process and the inference phase, and the models derived from training are generally larger in size and number of parameters, which are not favorable for mobile deployment^11^. In recent years, more and more scholars in the field of tea bud detection have achieved a lot of results in research model light-weighting. Cao et al.^12^ reduced the number of parameters of the model by replacing the normal convolution in the YOLOv5s model with the Ghost convolution, which has a precision of 76.31% with 5.94 M parameters and a model weight size of 10 MB. Zhang et al.^13^ replaced the backbone network of YOLOv5 with the ShuffleNetV2 network, and performed channel pruning on the neck layer and head network to reduce the model size, reducing the model size to 27% of the original model, with a size of 90.1 MB, but the mean average precision is reduced by 1.32%. Gui et al.^14^ introduced Ghost convolution into the YOLOv5l model to reduce the computing and model size of the model, and at the same time, added the bottleneck attention module in the backbone network and used weighted feature fusion in the neck network to improve the detection performance of the model, the improved model is smaller than the original model in terms of the number of parameters, computing, and model size, whose size are respectively 23.85 M, 56.89G, and 90 MB.

Inspired by the above research, this article further explores a lightweight tea bud detection model based on the YOLOv5 algorithm. While ensuring detection accuracy, the model has smaller size, parameters, and floating-point operations. To realize the above requirements this paper proposes a lightweight tea bud detection method with improved YOLOv5. The first step builds a standard YOLOv5s target detection model, and the second step replaces the backbone network of the model, CSPDarkNet-53, with the EfficientNetV2 network to drastically reduce the number of parameters and computing of the model. The third step utilizes the idea of GhostNet to eliminate the feature map redundancy, introduces the Ghost convolution in the neck network to replace the ordinary convolution, and constructs the C3Ghost module to replace the C3 module in the network to further reduce the size of the model. Finally, the CARAFE upsampling operator is introduced to replace the nearest Finally, the CARAFE upsampling operator is introduced to replace the nearest neighbor interpolation upsampling in the neck network, to improve the detection accuracy of the model for detecting tea buds. After a series of improvements and optimizations mentioned above, the number of parameters, floating-point operations, and model size of the YOLOv5 model has been greatly reduced, and at the same time, the accuracy of detecting tea buds can be guaranteed.

### Related work

Various studies^15,16^ have investigated the benefits of replacing the CSPDarkNet-53 backbone with the EfficientNet architecture, affirming that EfficientNet is an efficacious architecture that can be employed in visual detection tasks across diverse domains. This is owing to the inherent composite scaling method of EfficientNet, which generates a feature extraction network that is deeper, wider, and of higher resolution, significantly enhancing the model’s capacity to capture complex features and effectively enhancing the detection accuracy of the model. In addition, other studies^17–20^ have used low-cost computation to generate redundant feature mappings by introducing Ghost convolution instead of standard convolution in the neck network, thus reducing the parameters and computation of the model, demonstrating efficiency in computation and model size.

## Materials and methods

### Data acquisition

The tea bud images used in this study were collected in Shihe District, Xinyang City, Henan Province, the photographic data for the period from early April to early May 2023, the photographic time from 10:00 am to 2:00 pm, the photographic equipment is cell phones, the image size of 4000 × 3000 pixels, stored in JPG format. When collecting images of tea buds, the distance of the photographing equipment from the tea tree is 10 ~ 30 cm, and the angle between the photographing angle and the horizontal direction is between 0 ~ 60 degrees, and a total of 1889 original images of Xinyang Maojian buds were collected under natural illumination. Later on, the image data were manually screened to remove serious distortion, and finally, 1490 original image data of tea buds were obtained.

### Data set construction

To reduce the graphics memory consumption during the training process, the pixels of the 1490 tea bud images were first adjusted to a uniform size of 1280 × 960, and then the LabelImg annotation tool was used to annotate the tea bud images. In the LabelImg annotation tool use a rectangular box to box out all tea buds in the image, the labeling type is the bud, the content is set to bud, and the labeling information is saved as a TXT file^21^. During the labeling process, neither buds that are unclear or unsure, nor those with more than 50% obstruction, are tagged. To reduce the false detection rate and false recognition rate, and improve the generalization ability of the network model, 10 background images without tea buds were selected as negative sampling and were not annotated. They were combined with the annotated images to form the tea bud dataset.

We randomly selected 500 labeled images from the tea bud dataset as the test set, and the remaining 1000 labeled images were used as the training set and validation set. To improve the generalization ability of the trained model, 1,000 labeled images were subjected to a simple data enhancement process using a Python script program, including horizontal and vertical random flipping, contrast, and brightness random variation, to generate a total of 3,000 labeled images of tea buds. These labeled images are only used for the training process of the model, so they are divided into the training set and the validation set in the ratio of 8:2. The benefit of such a division of the dataset is that it ensures that there is no information leakage between the training set and the test set.

### Improving the model architecture of YOLOv5s

The YOLO series models are single-stage detection models, which are widely popular for their simple structure and balance between speed and accuracy and are used in various target detection tasks^22^. The YOLO series models are updated and iterated at a faster rate and have been updated to the YOLOv8 version, but the new version is still to be perfected. Therefore, this paper adopts the more mature YOLOv5 version, which also facilitates the deployment and acceleration of mobile processors. The network structure of the YOLOv5 target detection algorithm can be divided into three parts: the backbone network, the neck network, and the head network^23^. The backbone network of the YOLOv5 algorithm uses the structure of CSPDarknet-53 which is mainly used for feature extraction from the input image, the neck network uses a combination of feature pyramid network (FPN) and path aggregation network (PAN) to achieve multi-scale feature fusion^24^, and the head is used to make predictions of the features in three different dimensions to get the location and class of the detected object.

**Fig. 1:**
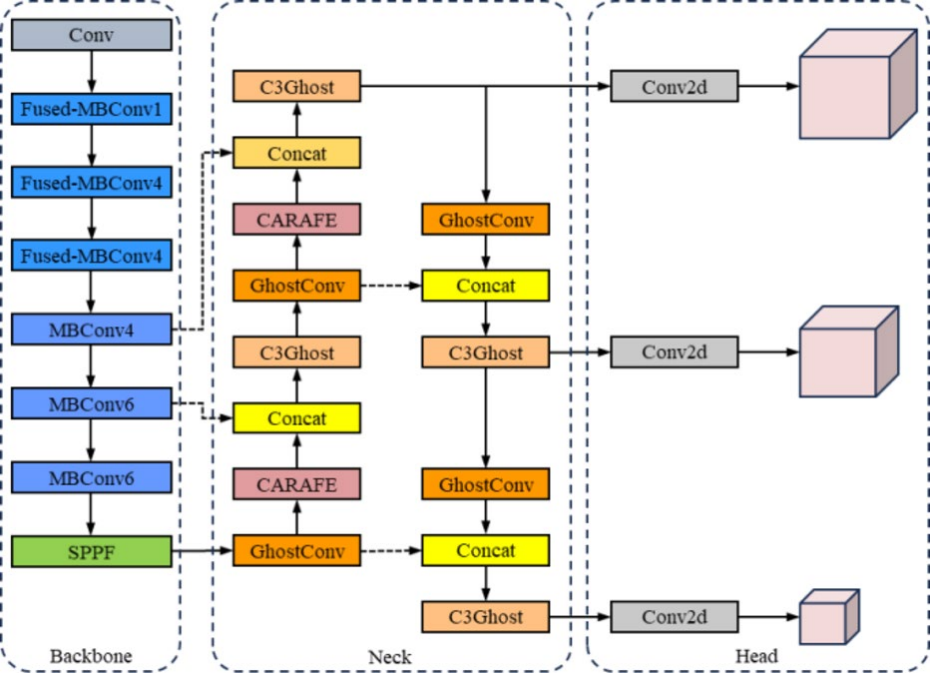
Improved tea bud detection model.

The YOLOv5 can be categorized into four versions, YOLOv5s, YOLOv5m, YOLOv5l, and YOLOv5x, according to the difference in network depth and width. In this study, the YOLOv5s model, which has a simpler network structure, is chosen as the basis for detecting tea buds, and at the same time, it is lightweight and improved to make it more suitable for deployment on mobile devices. The improved tea bud detection model is shown in Fig. 1. The model is mainly based on YOLOv5s to improve the backbone network and neck network to achieve the purpose of lightweight. Using the partial structure of the lightweight EfficientNetV2 network as a replacement for the backbone network of YOLOv5s, information on tea bud characterization is effectively extracted. The neck network of YOLOv5s retains the FPN + PAN structure, but ordinary convolutions and C3 modules are replaced with Ghost modules that construct Ghost convolutions and C3Ghost modules. This greatly reduces the number of redundant branches and further reduces the computing and total number of parameters of the model. To enhance the model’s ability to generalize in complicated situations, it is vital to consider the deep semantic knowledge of tea buds. Additionally, replace the nearest neighbor interpolation upsampling with the CARAFE upsampling operator in YOLOv5s. The CARAFE upsampling technique can combine contextual information of tea bud characteristics in a broader sensory field. This process can slightly increase the total number of parameters, while simultaneously boosting the model’s overall detection accuracy.

#### Improvement of the backbone network

EfficientNetV2 is a new smaller and faster convolutional neural network proposed by Mingxing Tan in 2021^25^. EfficientNetV2 proposes a training-aware neural architecture search (NAS), whose main search work is based on the EfficientNet network, removing unneeded search options as well as reusing the number of searched channels in EfficientNet as a way to reduce the size of the search space, while focusing on the joint optimization of accuracy, parameter efficiency, and training efficiency, and finally searched for the EfficientNetV2-S model architecture, as shown in Table 1. To accelerate the training speed, the authors propose an improved asymptotic learning method that dynamically adjusts the regularization approach based on the size of the training image and can improve the accuracy rate. EfficientNetV2 has the advantage of using parameters effectively while maximizing the training speed and improving the weaknesses of EfficientNetV1.

The architecture diagram of EfficientNetV2-S reveals that the network comprises eight stages, with the initial seven stages devoted to feature extraction for the detection task, while the final stage concerns classification. Therefore, this study requires the network structure from the first seven phases to replace the backbone network of YOLOv5s. Where Kernel represents the size of the convolution kernel, Stride represents the step size, Channels represents the channels of the feature matrix output from this stage, Expansion represents the rate of expansion of the input feature matrix channels, Layers represents the number of times the stage is repeatedly stacked, and Attention represents whether to use the squeeze and excitation attention module.

**Table 1:** EfficientNetV2-S architecture.

Stage	Operator	Kernel	Stride	Channels	Layers	Expansion	Attention
1	Conv	3 × 3	2	24	1	-	–
2	Fused-MBConv	3 × 3	1	24	2	1	–
3	Fused-MBConv	3 × 3	2	48	4	4	–
4	Fused-MBConv	3 × 3	2	64	4	4	–
5	MBConv	3 × 3	2	128	6	4	SE
6	MBConv	3 × 3	1	160	9	6	SE
7	MBConv	3 × 3	2	256	15	6	SE
8	Conv&Pooling&FC	1 × 1	–	1280	1	–	–

**Fig. 2:**
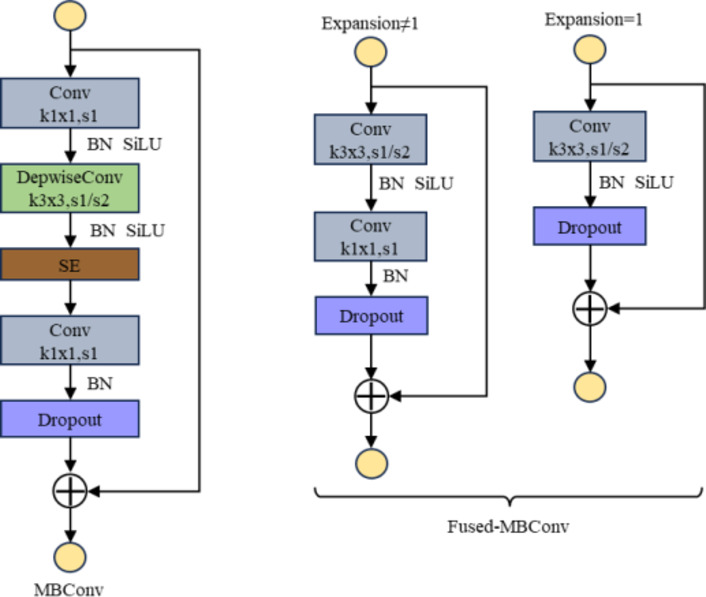
MBConv and Fused-MBConv structure.

According to Table 1, EfficientNetV2-S is mainly composed of the MBConv module and the Fused-MBConv module, and the detailed structures of the two modules are shown in Fig. 2. The MBConv module comprises five parts. The first part uses a 1 × 1 convolutional layer to expand the number of channels in the input feature matrix. The second part implements a 3 × 3 depthwise convolution that reduces the number of parameters in the model and the amount of computation. The third part is the SE Attention Module, which helps to enhance feature extraction from the model. The fourth part utilizes a 1 × 1 convolutional layer to reduce the number of channels in the feature matrix. Finally, the fifth part is the Dropout layer, which utilizes Stochastic Depth and has a probability of discarding the output from the main branch and using the output from the previous layer as the output for that layer. This reduces network depth and increases training speed. The Dropout layer and shortcut connection are only available when the stride is equal to 1 and the input and output feature matrix shapes are the same.

The Fused-MBConv module improves upon MBConv to benefit from mobile or server acceleration^26^. The Fused-MBConv structure is also very simple, it replaces conv1 × 1 and depthwise conv3 × 3 in the main branch of the original MBConv structure with a common conv3 × 3^27^. The Fused-MBConv structure is categorized into two types: one expands the number of input channels (Expansion ≠ 1), and the other does not (Expansion = 1). These two types differ in structure by a single 1 × 1 convolutional layer. Stochastic Depth is employed as a Dropout in both the Fused-MBConv module and the MBConv module, while the shortcut connections are used similarly.

#### Ghost model

**Fig. 3:**
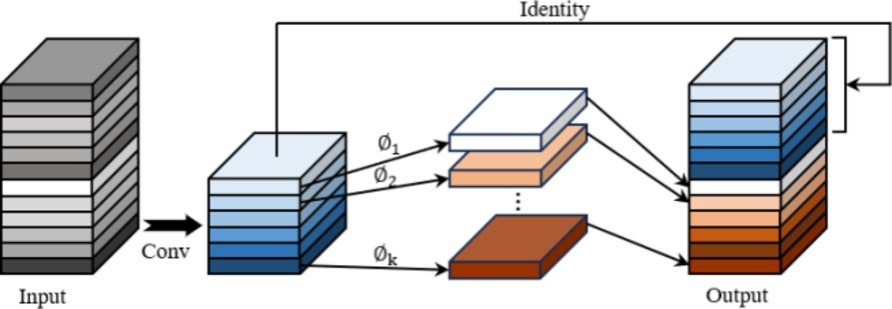
Ghost module.

The Ghost module is a lightweight convolutional module utilized in the GhostNet lightweight network that Han et al.^28^ proposed in 2020. The Ghost module uses a series of linear transformations based on a set of intrinsic feature maps to extract the desired information from the original features at a very low cost. The total number of parameters and computational complexity of ghost convolution is reduced compared to normal convolution, as shown in Fig. 3 for the working principle of the Ghost module. From the figure, the process can be divided into three parts, the first part is an ordinary convolution operation to generate some intrinsic feature maps with a small number of 1 × 1 convolutions, and the second part generates ghost feature maps by performing a series of cheap linear operations on the intrinsic feature maps generated in the first part. Finally, the feature maps generated in the first two parts are concatenated together by channel number and transferred to the subsequent network.

Assuming that the size of the input feature map is $\:h\times\:w\times\:c\:$, the size of the output feature map is $\:{h}^{{\prime\:}}\times\:{w}^{{\prime\:}}\times\:n\:$, and the size of the ordinary convolution kernel is *k*, the ordinary convolution floating-point operations(FLOPs) is calculated as follows:

1 $$\:f=n\cdot\:{h}^{{\prime\:}}\cdot\:{w}^{{\prime\:}}\cdot\:c\cdot\:k\cdot\:k$$

The Ghost module FLOPs is calculated as follows:

2 $$\:{f}^{{\prime\:}}=\frac{n}{s}\cdot\:{h}^{{\prime\:}}\cdot\:{w}^{{\prime\:}}\cdot\:c\cdot\:k\cdot\:k+(s-1)\cdot\:\frac{n}{s}\cdot\:{h}^{{\prime\:}}\cdot\:{w}^{{\prime\:}}\cdot\:d\cdot\:d$$

In Eq. 2, *d* is the linearly transformed convolutional kernel size and *s* represents the total mapping produced by each channel. In general, $\:s\ll\:c,\:d\approx\:k$. The theoretical speed-up ratio of ordinary convolution replaced by ghost module is as follows:

3 $$\:\frac{f}{{f}^{{\prime\:}}}=\frac{n\cdot\:{h}^{{\prime\:}}\cdot\:{w}^{{\prime\:}}\cdot\:c\cdot\:k\cdot\:k}{\frac{n}{s}\cdot\:{h}^{{\prime\:}}\cdot\:{w}^{{\prime\:}}\cdot\:c\cdot\:k\cdot\:k+(s-1)\cdot\:\frac{n}{s}\cdot\:{h}^{{\prime\:}}\cdot\:{w}^{{\prime\:}}\cdot\:d\cdot\:d}\approx\:\frac{s\cdot\:c}{s+c-1}\approx\:s$$

It can be inferred from Eq. 3 that the FLOPs needed in the ghost module have decreased when the size of the output feature maps remains unchanged, compared to the ordinary convolution. This decrease is equivalent to about 1/s of the ordinary convolution.

**Fig. 4:**
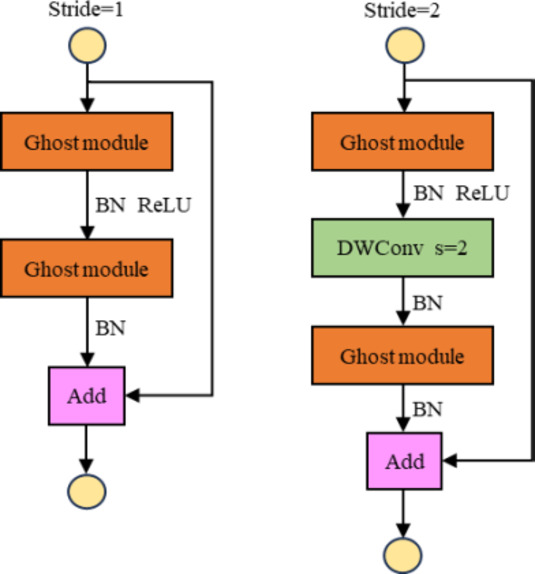
Ghost bottleneck structure.

Han et al. designed the Ghost bottleneck structure by referring to the ResNet^29^ residual module, as shown in Fig. 4. The ghost bottleneck consists of two stacked Ghost modules^30^. The first Ghost module is used to increase the number of channels of the input feature map. The second Ghost module is used to reduce the number of channels of the feature map to the same number of channels as the input feature map. Then use a shortcut to connect the inputs and outputs of the two Ghost modules. When stride = 2, a depthwise convolution with a step of 2 is added between the two Ghost modules.

**Fig. 5:**
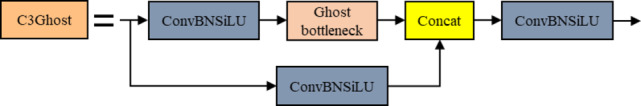
C3Ghost module structure.

The C3Ghost module structure, shown in Fig. 5, is created by combining the C3 module with the ghost bottleneck. The benefit of this design is that it can significantly decrease the computational complexity and the number of parameters of the model during the convolution operation without destroying the structure of the C3 module.

#### CARAFE upsampling operator

In current convolutional neural networks, the operation of feature upsampling plays a pivotal role. In the original YOLOv5, the nearest neighbor interpolation was used for upsampling. The nearest neighbor interpolation only up-samples feature points based on their spatial locations, ignoring valuable semantic information extracted from the feature map. This approach reduces the quality of the up-sampled feature map. Therefore, to obtain a higher quality up-sampled feature map, the lightweight upsampling operator CARAFE is utilized in place of nearest neighbor interpolation. CARAFE upsampling possesses a wide receptive field for gathering contextual feature information, instance-specific content-aware processing capabilities, and the capacity to dynamically generate adaptive kernels, as well as the benefits of being both lightweight and computationally efficient^31^.

CARAFE consists of two components: a kernel prediction module and a content-aware reassembly module^32^. In the kernel prediction module, the channels of the input feature map are first compressed using a $\:1\times\:1$ convolution, and then the compressed feature map is encoded using a coding kernel to generate a reorganization kernel, while the channels of the feature map are then unrolled in the spatial dimension. Finally, the recombination kernel is normalized through the softmax function to ensure that the weights of the convolution kernel sum to 1. In the content-aware reassembly section, each position on the output feature map is mapped one-to-one with the input feature map, and the output value is obtained by taking the $\:k\times\:k$ region of the input feature map and doing the dot product operation with the predicted upsampling kernel, and different channels at the same position share a single upsampling kernel.

### Experimental process

#### Experimental environment

The arithmetic resource used in this study is the AutoDL arithmetic cloud platform, and the CPU is Intel Xeon Platinum 8350 C with 16 cores, 56GB of running memory, and the GPU is NVIDIA GeForce RTX3090 24G. The operating system is Ubuntu 18.04 with the PyTorch deep learning framework, PyTorch version 1.9.0, Cuda version 11.1, Torchvision version 0.10.0, and Python version 3.8.

#### Experimental details

The model training parameters are set as follows: YOLOv5 version 7.0, the initial learning rate is 0.01, the momentum factor is 0.937, the learning rate decline function is cosine function, the model optimizer is SDG, the optimizer weight decay factor is 0.0005, the input image size is set to 640 × 640 pixels, the batch size is set to 16, and Mosaic data enhancement is used, the total number of training epochs for the data obtained in this study are all 400.

#### Experimental indicators

The study uses several evaluation indicators: mean average precision(mAP), precision(P), and recall(R) are used to measure the accuracy of the model training and the performance of the model detection. Parameters, floating point operations (FLOPs), and weights are used to measure the complexity of the trained model. The formulas for the expression of precision, recall, and mean average precision are as follows:

4 $$\:P=\frac{TP}{TP+FP}$$

5 $$\:R=\frac{TP}{TP+FN}$$

6 $$\:mAP=\frac{1}{N} \sum \limits_{i=1}^{N}AP=\frac{1}{N} \sum \limits_{i=1}^{N}{\int\:}_{0}^{1}P\left(R\right)dR$$

TP is true positive, indicating the number of actual positive samples are predicted to also be positive samples. FP is false positive, indicating the number of samples that are actual negative samples but are predicted to be positive. FN is false negative, indicating the number of samples that are positive but predicted to be negative samples. Precision is the proportion of actual positive samples to all predicted positive samples, reflecting the accuracy of the model’s detection. Recall refers to the proportion of predicted positive samples to actual positive samples, reflecting the missed detection ability of the model. AP is the average precision, where N is the number of categories, in this study there is only one category of tea buds, so *N* = 1 and the average precision is equal to the mean average precision.

## Experiments and discussion

### Experimental comparison of different backbone networks

This experiment utilizes the YOLOv5s target detection model, trained using various backbone networks including ShuffleNetV2^33^, PP-LCNet^34^, MobileNetV3^35–37^, GhostNetV1, and EfficientNetV2, while keeping other parameters unchanged. The parameters obtained are analyzed and compared in Table 2 to verify the feasibility of the improved model.

**Table 2:** Performance comparison of different backbone networks.

Backbone network	*P*/%	*R*/%	$\:{\text{m}\text{A}\text{P}}_{0.5}$/%	Parameter/M	FLOPs/G	Weights/MB
CSPDarkNet-53	79.48	81.24	84.12	7.01	15.76	13.8
ShuffleNetV2	75.89	74.95	78.95	4.44	8.50	8.9
PP-LCNet	79.17	66.25	74.43	4.40	8.62	8.8
MobileNetV3	79.62	66.46	74.92	3.75	6.45	7.6
GhostNetV1	76.36	73.06	78.09	7.23	10.32	14.4
EfficientNetV2	82.35	77.04	84.44	5.40	5.58	10.6

The experimental comparison of improved backbone networks is conducted on the self-constructed dataset of this study. As shown in Table 2, when using the EfficientNetV2 network as the backbone network of YOLOv5s for tea bud feature extraction, the model parameters amount to 5.40 M, with the floating-point operations of 5.58G, and the weights size of 10.6 MB. The detection accuracy achieved is 82.35%, the recall is 77.04%, and the mean average precision is 84.44%. Compared to the original model that used the CSPDarkNet-53 backbone network, the improved backbone network has 22.97% fewer model parameters and 64.59% fewer floating-point operations, as well as the mean average precision improvement of 0.32% points and the detection precision improvement of 2.87% points, while reducing the weight by 3.2 MB. According to the experimental results, the detection performance of the model after changing the backbone structure to the EfficientNetV2 network is better than the unimproved model. Compared to other lightweight backbone networks ShuffleNetV2, PP-LCNet, MobileNetV3, and GhostNetV1, the EfficientNetV2 network improves the mean average precision by 5.49, 10.01, 9.52, and 6.35% points, and reduces the floating-point operations by 2.92G, 3.04G, 0.87G, and 4.74G, respectively. Although the ShuffleNetV2, PP-LCNet, and MobileNetV3 networks have fewer parameters and a smaller model weight compared to EfficientNetV2, for the tea bud detection task, it is essential to prioritize the average detection precision. Therefore, it is necessary to ensure that the overall detection precision does not decrease while reducing the parameters and computational complexity of the model as much as possible. In brief, selecting the EfficientNetV2 network as the backbone structure not only elevates the model’s detection accuracy but also reduces the parameters and the floating-point operations, thus significantly alleviating mobile deployment’s pressure.

### Model ablation experimental studies

Based on the data presented in Table 2, it can be concluded that utilizing the EfficientNetV2 network as the backbone of YOLOv5s to extract tea bud features results in improved detection performance. In this study, the EfficientNetV2 network is substituted for the backbone network of YOLOv5. Further optimization of the neck network is achieved by introducing the Ghost module and the CARAFE upsampling operator. The ablation study is performed to verify whether the improvements can improve the model performance. According to Table 3, it can be concluded that when the ghost module alone is introduced to replace the normal convolution and C3 module in the neck network, the parameters are reduced by 25.92%, the floating-point operations is reduced by 11.29%, and the size of the weights is reduced by 24.53%. By aggregating feature information over a larger sensory field, CARAFE upsampling can effectively enhance the model detection accuracy for tea buds. According to Table 3, introducing only CARAFE upsampling resulted in a 2.2% improvement in model precision, 0.2% improvement in model recall, and 1.1% improvement in mean average precision. Similarly, the CARAFE upsampling operator results in a slight increase in parameters and floating-point operations. This can be attributed to the use of distinct up-sampling kernels for sampling different feature layers. The results of the ablation experiments show that optimizing the neck network can achieve the expected results, provided that the backbone network is replaced by the EfficientNetV2 network.

**Table 3:** Ablation experiment results.

Methods	*P*/%	*R*/%	$\:{\text{m}\text{A}\text{P}}_{0.5}$/%	Parameters/M	FLOPs/G	Weights/MB
EfficientNetV2	82.35	77.04	84.44	5.40	5.58	10.6
EfficientNetV2 + Ghost	85.50	76.10	84.61	4.00	4.95	8.0
EfficientNetV2 + CARAFE	84.51	77.20	85.46	5.54	5.65	10.9
EfficientNetV2 + Ghost + CARAFE	83.50	77.46	85.79	4.14	5.02	8.3

### Comparison of the model before and after improvement

**Fig. 6:**
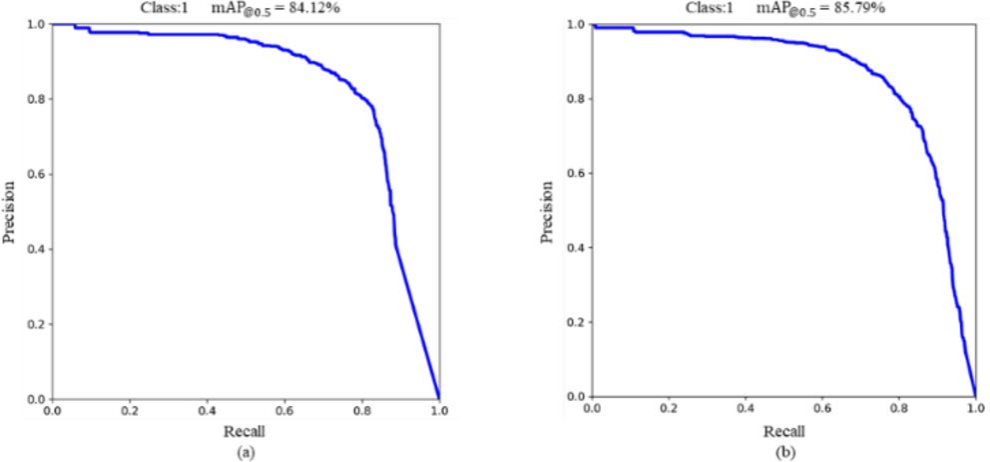
Precision-Recall curve before (**a**) and after (**b**) improvement.

**Fig. 7:**
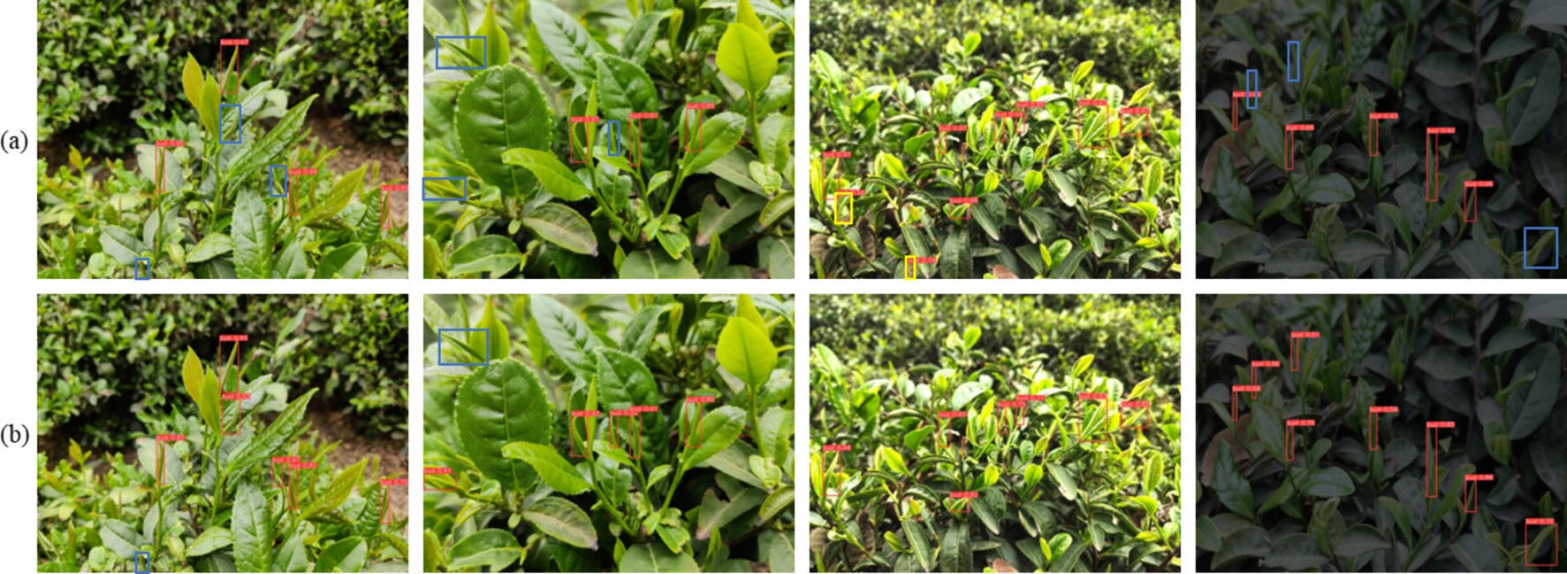
Detection effect before (**a**) and after (**b**) model improvement. Yellow box indicates detection error, blue box indicates missed detection.

The experimental comparisons of the models before and after the improvements are conducted on the self-constructed dataset. From the information presented in, the improved model increased precision by 4.02%, increased mAP by 1.67%, reduced parameters by 40.94%, reduced FLOPs by 68.15%, and reduced model weights by 39.86%. The mAP is a combined evaluation metric of the model precision and recall that can be used to measure the detection performance of the model. From Eq. 6, it can be inferred that the mAP value is equivalent to the PR curve and the axis’s enclosed area. In combination with Fig. 6, it can also be inferred that the mAP value of the improved model is slightly higher than that of the original model. Although the recall of the improved model decreased by 3.78%, the model detection performance improved. FPS is used to measure the inference speed of the model. From Table 4, it is apparent that while the improved model’s inference speed has decreased, the detection frame rate of 85 frames per second is sufficient for real-time harvesting purposes. The results of the experiment demonstrate that the improved model effectively reduces the parameters and FLOPs and reduces the computational cost compared to the original model. Whilst the performance of the detection is enhanced, the storage capacity necessitated by the model is efficaciously diminished. To compare the detection effect before and after the improvement, four randomly selected (simple and complex backgrounds, strong light and dark light) pictures are tested and the results are shown in Fig. 7. The detection capability of the improved model is better than the unimproved model in several scenarios.

**Table 4:** Comparison of the model performance before and after improvement.

Model	*P*/%	*R*/%	$\:{\text{m}\text{A}\text{P}}_{0.5}$/%	Parameters/M	FLOPs/G	Weights/MB	Speed/FPS
YOLOv5s	79.48	81.24	84.12	7.01	15.76	13.8	167
Ours	83.50	77.46	85.79	4.14	5.02	8.3	85

###  Experimental comparison of different detection algorithms

The improved lightweight model is compared to other detection algorithm models to analyze their detection performance and explore the superiority of the improved algorithm. The study compares the improved tea bud detection model based on YOLOv5s with the current mainstream YOLO algorithms and presents the experimental findings in Table 5.

**Table 5:** Results of different detection algorithms for tea buds.

Models	$\:{\text{m}\text{A}\text{P}}_{@0.5}$/%	Parameters/M	FLOPs/G	Weights/MB
YOLOv3-tiny	78.76	8.67	12.88	16.6
YOLOv4	76.13	52.46	118.88	100.6
YOLOv5s	84.12	7.01	15.76	13.8
YOLOv6s	82.64	16.30	44.03	31.3
YOLOv7-tiny	80.85	6.01	13.02	11.7
YOLOv8s	83.80	11.13	28.44	21.5
YOLOX-s	85.40	8.94	26.76	68.5
Ours	85.79	4.14	5.02	8.3

The study results indicate that the YOLOv4 network model is unsuitable for tea bud detection and identification due to its higher number of parameters and floating-point operations, large weights, and low mAP value. The YOLOX-s network model obtained high mAP values on this dataset, with mAP values only 0.39% lower than the improved model, but there is the problem of larger model weights. When comparing the YOLOv5s model to other models of similar size, it was found that the YOLOv5s model had the best detection accuracy on the self-constructed dataset, as well as smaller parameters, FLOPs, and weights. Therefore, it is the best choice to improve the lightweight based on the YOLOv5s model, the improved model has the highest detection accuracy, the improved model has the smallest number of parameters and FLOPs, and the weight of the improved model is also the smallest, and the mAP value for the detection of tea buds reaches 85.79%.

## Conclusions

When utilizing the tea bud detection and recognition model in the actual picking task, the detection accuracy and the calculation and size of the model need to be considered. In the future, it is essential to investigate the lightweight tea bud detection model, which will be deployed to mobile and used to relieve structural pressure on the tea-picking robot. Based on actual tea garden picking scenarios, several modifications were implemented to the YOLOv5s model. The YOLOv5s backbone network now uses EfficientNetV2, and the Ghost module replaces the conventional convolution and C3 module in the neck network. These changes reduce model size and computation. To enhance the model’s detection accuracy, we simultaneously introduce the CARAFE upsampling operator. Compared to YOLOv5s, the improved model has 40.94% fewer parameters, 68.15% fewer FLOPs, and 39.86% lighter weights. The detection precision, recall, and mAP of tea buds under different light conditions were 83.50%, 77.46%, and 85.79%, respectively. Compared to other popular detection models in the YOLO series, the improved model stands out due to its smaller computation and model size, higher detection accuracy, and ability to meet the real-time picking requirements.

This study provides a new and improved method for lightweight tea bud detection models and achieves better results on the self-constructed dataset. The model in this study can detect single tea buds but cannot grade them. Future work should optimize the model to detect tea buds of different grades and improve applicability to different seasons.

## Data Availability

The raw and processed data required to reproduce these results are available by contacting the author Bohan Yuan.
